# Infection episodes and islet autoantibodies in children at increased risk for type 1 diabetes before and during the COVID-19 pandemic

**DOI:** 10.1007/s15010-024-02312-y

**Published:** 2024-06-14

**Authors:** Ivo Zeller, Andreas Weiss, Stefanie Arnolds, Katharina Schütte-Borkovec, Sari Arabi, Thekla von dem Berge, Kristina Casteels, Angela Hommel, Olga Kordonouri, Helena Elding Larsson, Markus Lundgren, Anne Rochtus, Matthew D. Snape, Agnieszka Szypowka, Manu Vatish, Christiane Winkler, Ezio Bonifacio, Anette-Gabriele Ziegler, for the GPPAD Study Group

**Affiliations:** 1 Institute of Diabetes Research, Helmholtz Munich, German Center for Environmental Health, Heidemannstrasse 1, 80939 Munich, Germany; 2 Center for Regenerative Therapies Dresden, Technische Universität Dresden, Dresden, Germany; 3 Department of Pediatrics, University Hospital Carl Gustav Carus, Technische Universität Dresden, Dresden, Germany; 4 Kinder- Und Jugendkrankenhaus AUF DER BULT, Hannover, Germany; 5 Department of Pediatrics, University Hospitals Leuven, Louvain, Belgium; 6 Department of Development and Regeneration, KU Leuven, Louvain, Belgium; 7 Unit for Pediatric Endocrinology, Department of Clinical Sciences Malmö, Lund University, Lund, Sweden; 8 Department of Paediatrics, Skåne University Hospital, Malmö/Lund, Sweden; 9 Department of Pediatrics, Kristianstad Hospital, Kristianstad, Sweden; 10 Oxford Vaccine Group, University of Oxford Department of Paediatrics, NIHR Oxford Biomedical Research Centre, Oxford, UK; 11 Department of Paediatrics, Medical University of Warsaw, Warsaw, Poland; 12 Nuffield Department of Women’s & Reproductive Health, Oxford, UK; 13 Paul Langerhans Institute Dresden of the Helmholtz Munich at University Hospital Carl Gustav Carus, Faculty of Medicine, TU, Dresden, Germany; 14 Forschergruppe Diabetes E.V. at Helmholtz Munich, German Research Center for Environmental Health, Munich, Germany; 15 Forschergruppe Diabetes, School of Medicine, Klinikum Rechts Der Isar, Technical University Munich, Munich, Germany

**Keywords:** Infection, Type 1 diabetes, Autoimmunity, COVID-19

## Abstract

**Objectives:**

To determine the impact of the COVID-19 pandemic on the incidence rates of infection and islet autoimmunity in children at risk for type 1 diabetes.

**Methods:**

1050 children aged 4 to 7 months with an elevated genetic risk for type 1 diabetes were recruited from Germany, Poland, Sweden, Belgium and the UK. Reported infection episodes and islet autoantibody development were monitored until age 40 months from February 2018 to February 2023.

**Results:**

The overall infection rate was 311 (95% Confidence Interval [CI], 304–318) per 100 person years. Infection rates differed by age, country, family history of type 1 diabetes, and period relative to the pandemic. Total infection rates were 321 per 100 person-years (95% CI 304–338) in the pre-pandemic period (until February 2020), 160 (95% CI 148–173) per 100 person-years in the first pandemic year (March 2020—February 2021; *P* < 0.001) and 337 (95% CI 315–363) per 100 person-years in subsequent years. Similar trends were observed for respiratory and gastrointestinal infections. Islet autoantibody incidence rates were 1.6 (95% CI 1.0–2.4) per 100 person-years in the pre-pandemic period, 1.2 (95% CI 0.8–1.9) per 100 person-years in the first pandemic year (*P* = 0.46), and 3.4 (95% CI 2.3–4.8) per 100 person-years in subsequent years (*P* = 0.005 vs. pre-pandemic year; *P* < 0.001 vs. first pandemic year).

**Conclusions:**

The COVID-19 pandemic was associated with significantly altered infection patterns. Islet autoantibody incidence rates increased two-fold when infection rates returned to pre-pandemic levels.

**Supplementary Information:**

The online version contains supplementary material available at 10.1007/s15010-024-02312-y.

## Introduction

Infections are believed to contribute to genetic diversity, thereby influencing susceptibility for immune-mediated diseases [1]. Type 1 diabetes is a chronic autoimmune disease characterized by the destruction of insulin-producing beta cells in the pancreas, leading to insulin deficiency. Islet autoimmunity precedes clinical type 1 diabetes and often manifests in the first three years of life [2]. In support of the role of infection in the etiology of type 1 diabetes, various viral response genes have been identified as conferring susceptibility to the disease. Moreover, numerous reports indicate associations between infection and the development of islet autoimmunity in children who have a prior genetic susceptibility, particularly in the first years of life [3–9].

During the COVID-19 pandemic, infections underwent significant changes [10]. A novel virus was introduced to the human population and, within less than three years, had likely affected the majority of individuals [11]. Additionally, preventive measures implemented during the pandemic have altered healthcare-seeking behavior, patterns of infectious disease transmission, and the incidence of infectious episodes. The pandemic was directly associated with an increase in type 1 diabetes incidence [12–14] and infection with the SARS-CoV-2 virus in early childhood was further associated with the risk of developing islet autoimmunity [9].

The aim of this study was to assess the exposure rate and potential changes in infection exposures by age within the first 3 years of life. The study also sought to determine whether there was a change in infection rates from the pre-pandemic to post-pandemic periods and whether these were associated with incidence rates of islet autoimmunity. This investigation was performed in the context of a clinical trial in 1050 children with a genetic susceptibility for type 1 diabetes who were monitored with adverse event reporting from infancy. The findings carry implications for understanding the impact of pandemics on the infection landscape and immune mediated diseases.

## Methods

### Participants

The study was performed in 1050 children participating in the Primary Oral Insulin Trial (POInT). POInT investigates whether daily intake of oral insulin reduces the incidence of islet autoimmunity and/or type 1 diabetes in children with an increased risk of type 1 diabetes [15]. Children were eligible if they had an increased risk for developing islet autoimmunity of > 10% by the age of 6.0 years, defined by a genetic risk score [16] (Supplementary Table S1). Enrollment commenced in February 2018 and ended in March 2021. Children were enrolled at the age of 4.0–7.0 months, and followed at 2, 4, and 8 months after study enrollment, at 1.5 years of age, and then every 6 months. Daily treatment with oral insulin or placebo continued until age 3 years. The study was conducted in seven clinical research centers including three in Germany (Dresden, Hanover, and Munich), one in Sweden (Malmö), one in Poland (Warsaw), one in Belgium (Leuven), and one in the UK (Oxford). Adverse events were recorded at each clinical study visit until 6 months after end of treatment. For the current analysis, reported infections until 02/28/2023 were included. A detailed description of the study protocol has been published previously [15].

### Assessment of exposure to infections

At each scheduled clinic visit, adverse events were collected by study personnel and recorded in the clinical trial database. For each event, illness description, date of onset and end date was recorded. Adverse events were categorized using the Medical Dictionary for Regulatory Activities (MedDRA). Classification of infections was performed as previously described [17] (Supplementary Table S2). In brief, the first infection episode category was created from MedDRA Lowest Level Terms (LLT) as respiratory infection. If terms belonging to this category were reported within one week, they were regarded as one respiratory infection episode. Gastrointestinal symptoms are considered to occur frequently in young children with a respiratory infection and therefore gastrointestinal symptoms co-occurring with respiratory infections were considered as part of the respiratory infection episode. The second infection episode category was created from MedDRA LLT as gastrointestinal infections. If terms belonging to this category were reported within the same week, they were regarded as one gastrointestinal infection episode. The third category was defined as “other types of infections”, and the fourth category was unknown febrile episodes. Each LLT within this category is treated as a distinct infection episode. The date of all episodes was the date of the first LLT. A separate category was given to infections associated with Coxsackie virus (Supplementary Table S2). In the present study, follow-up data up to 40 months of age were evaluated.

### Islet autoimmunity outcome

Islet autoantibodies were measured centrally at two independent GPPAD Core laboratories, located at the Institute of Diabetes Research, Helmholtz Munich, Germany, and at the University of Bristol Medical School, Diabetes and Metabolism, Learning and Research, Southmead Hospital, Bristol, United Kingdom (for confirmation of results). Serum samples from each visit were analyzed for autoantibodies to insulin, GAD65, IA-2 and ZnT8 (ZnT8RA and ZnT8WA) as previously described [18]. A child was classified as islet autoantibody positive if 2 consecutive samples tested positive at both laboratories. A child was classified as multiple islet autoantibody positive if tested positive for 2 or more autoantibodies in both laboratories. The islet autoimmunity outcome was defined as either development of multiple islet autoantibodies or the development of one or more islet autoantibodies followed by type 1 diabetes. Maternally transferred islet autoantibodies were excluded and identified if the child was positive at the first sample, had declining antibody titers on follow-up, and subsequently became islet autoantibody-negative. For children classified as islet autoantibody-positive, the first positive sample was taken as the age at seroconversion.

### Study approval

Ethical approval for the POInT study was obtained from local ethical committees and regulatory authorities of the Technische Universität München, Medical Faculty (326/17 Af), the Medical University of Warsaw (Institute of Mother and Child) (199/2017), the UK Health Research Authority (18/SC/0019), Onderzoek UZ/KU Leuven (S60711) and the Regionala etikprövningsnämnden i Lund (2017/918). The parents or legal representatives of each participant provided written informed consent, and further agreed to biobank storage of material that was used in this study.

### Statistical analysis

Age- and stage-specific counts of infection episodes were calculated across distinct age intervals: 4–8.99 months, 9–14.99 months, 15–20.99 months, 21–26.99 months, 27–32.99 months, and 33–39.99 months. Infection episode counts were also segmented into pre-pandemic (2018–02-07 to 2020–02-29), pandemic 2020 (2020–03-01 to 2021–02-28), and pandemic 2021–2022 (2021–03-01 to 2023–02-28) periods. Infection episodes for each group combination were expressed as infection rates per 100 person-years.

Multivariable Poisson regressions incorporating sex, HLA risk group, GP/FDR status, country, age group and pandemic stage as covariates were applied to model the various infection rates over a maximum 36-month period. The significance of each categorical variable was assessed using Wald tests, comparing each category against its respective reference. Results were expressed as rate ratios (with 95% confidence intervals) or as a percentage change in the rate.

To facilitate specific pairwise comparisons among age groups and stages of the pandemic, contrast matrices were developed and employed. Given that these pairwise comparisons entailed conducting multiple tests, the Bonferroni adjustment was applied to account for this multiplicity.

In the study of islet autoantibody incidence, rate ratios were calculated as the ratio derived from the calculated incidences across different groups. To assess the statistical significance of differences between these rate ratios a proportion test was performed based on the chi-squared statistic.

Throughout the study, statistical significance was determined based on p-values being less than 0.05. Graphs were generated using the ggplot2 package (version 3.4.4), and all statistical analyses were conducted using R software (version 4.3.2, https://www.R-project.org/).

## Results

A comprehensive analysis of infection episodes was conducted longitudinally, covering the period from age 4 months to 40 months in the 1050 enrolled children (Table 1). The participants included 80 children from Belgium, 504 from Germany, 242 from Poland, 173 from Sweden, and 51 from UK. Enrollment commenced in February 2018 and ended in March 2021. Follow-up for the current analysis ended in February 2023. The cumulative observation time was 2422 person-years (Table 1). The total number of infection episodes was 7525, including 5237 (70%) respiratory infection episodes, 767 (10%) gastrointestinal infection episodes, 593 (8%) other classified infection episodes and 928 (12%) unknown febrile episodes (Table 1).

**Table 1:** Infection Episodes in Children Aged 4 to 40 months

Country	N	Observation time (person years)	Total infection Episodes	Respiratory infections *n* (%)	Gastro-intestinal infections *n* (%)	Other infections *n* (%)	Unknown febrile episodes *n* (%)
Belgium	80	188	636	412 (65%)	56 (9%)	66 (10%)	102 (16%)
Germany	504	1148	3448	2315 (67%)	445 (13%)	223 (6%)	465 (13%)
Poland	242	570	1506	1066 (71%)	104 (7%)	220 (15%)	116 (8%)
Sweden	173	404	1810	1371 (76%)	147 (8%)	55 (3%)	237 (13%)
UK	51	112	125	73 (58%)	15 (12%)	29 (23%)	8 (6%)
Total	1050	2422	7525	5237 (70%)	767 (10%)	593 (8%)	928 (12%)

The overall infection incidence rate was 311 (95% CI 304–318) episodes per 100 person-years. Incidence rate was influenced by age, increasing from 240 (95% CI 222–260) per 100 person-years in children aged 4 to 9 months to a peak of 318 (95% CI 289–349) per 100 person-years in children aged 15 to 21 months (*P* < 0.001; Table 2). The overall incidence rate of respiratory infections was 216 (95% CI 210–222) per 100 person-years and the overall rate of gastrointestinal infections was 32 (95% CI 29–34) per 100 person years with similar age trends. The overall rate of infections attributed to Coxsackie virus, which has been associated with the development of islet autoimmunity, was 9 (95% CI 8–10) per 100 person-years.

**Table 2:** Influence of age, sex, country, genetics and period on total infection rates

Covariate	N	Adj. Infection Rate (95% CI)	Infection Episode Rate Ratio (RR)		
			RR	95% CI	*P* value
Age Group					
4–9 months	1050	240 (222–260)	1.00	reference	0.11
9–15 months	1035	258 (234–284)	1.08	0.98–1.19	< 0.001
15–21 months	1016	318 (289–349)	1.33	1.21–1.46	< 0.001
21–27 months	1003	304 (275–337)	1.27	1.15–1.41	< 0.001
22–33 months	983	282 (256–315)	1.18	1.07–1.32	0.002
33–40 months	787	292 (261–330)	1.22	1.09–1.38	< 0.001
Sex					
Female	519	307 (297–317)	1.00	reference	
Male	531	313 (301–359)	1.02	0.98–1.17	0.33
Country					
Germany	504	300 (290–311)	1.00	reference	
Belgium	80	321 (294–359)	1.07	0.98–1.17	0.10
UK	51	114 (96–135)	0.38	0.32–0.45	< 0.001
Poland	242	267 (249–282)	0.89	0.83–0.94	< 0.001
Sweden	173	445 (418–472)	1.48	1.39–1.57	< 0.001
Family History with Type 1 Diabetes					
Yes	555	292 (283–302)	1.00	reference	
No	495	325 (30–348)	1.11	1.03–1.19	0.005
HLA genotype					
DR3/DR4-DQ8	565	278 (258–299)	1.00	reference	
DR4-DQ8/DR4- DQ8	95	286 (264–311)	1.03	0.95–1.12	0.46
Other genotypes	390	297 (275–322)	1.07	0.99–1.16	0.07
Period					
Pre-Pandemic	583	321 (304–338)	1	reference	
Pandemic 2020	973	160 (148–173)	0.50	0.46–0.54	< 0.001
Pandemic 2021–2022	941	337 (315–363)	1.05	0.98–1.13	0.15

In addition to age, the infection incidence rate was affected by site, the family history of type 1 diabetes and the pandemic period, but not sex or HLA genotype (Table 2). The adjusted rate of reported infections across the whole age range was highest in Sweden (445 per 100 person-years; 95% CI 418–472) and lowest in the UK (114 per 100 person-years; 95% CI 96–135). The adjusted infection rate was higher in children without a first-degree family history of type 1 diabetes (325 per 100 person-years; 95% CI 301–348) compared to children with a first-degree family history of type 1 diabetes (292 per 100 person-years; 95% CI 283–302; *P* = 0.005).

### Infection rates in relation to the COVID-19 pandemic

**Fig. 1:**
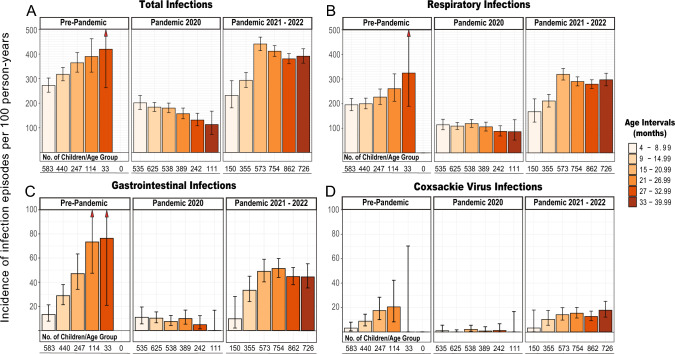
Incidence of Any Infection Episodes in Children at Increased Risk for Type 1 Diabetes Before and During the COVID-19 Pandemic The unadjusted incidence rates of total infection episodes **(A)**, respiratory infections **(B)**, gastrointestinal infections **(C)**, and Coxsackie virus-associated infections **(D)** are shown for the period 2018–02-07 to 2020–02-29 (pre-pandemic), 2020–03-01 to 2021–02-28 (pandemic 2020), and 2021–03-01 to 2023–02-28 (pandemic 2021–2022). Infection episodes are expressed as incidence rate per 100 person-years, and are presented across distinct age intervals (see color-codes): 4–8.99 months, 9–14.99 months, 15–20.99 months, 21–26.99 months, 27–32.99 months, and 33–39.99 months. The number of children in each age interval is given below the respective bar

The Poisson model incidence rate of infections decreased by 50% from 321 (95% CI 304–338) per 100 person-years in the period 2018 to February 2020 to 160 per 100 person-years (95% CI 148–173) per 100 person-years (*P* < 0.001) during the first 12 months of the pandemic (March 2020 to February 2021) (Table 2, Fig. 1; Supplementary Table S3). The decrease was observed for each age group (adjusted *P* < 0.001) and specifically for respiratory infections with a 54% decrease (216 per 100 person-years; 95% CI 210–222 vs. 99 per 100 person years; 95% CI 91–110; *P* < 0.001) and gastrointestinal infections with a 76% decrease (32 per 100 person-years; 95% CI 29–34 vs. 8 per 100 person years; 95% CI 5–10; *P* < 0.001). The incidence rate of infections attributed to Coxsackie virus infections reduced dramatically by 92% from 9 per 100 person years (95% CI 8–10) pre-pandemic to 1 (95% CI 0–2) during the first pandemic year (*P* < 0.001). Decreases of any infectious episodes were observed in each country with the least variation observed in Poland (adjusted reduction, 20%) and Sweden (adjusted reduction, 28%) and over 45% reduction in each of Germany, the UK, and Belgium (Supplementary Table S4). The association between elevated total and specific infection rates and advancing age was no longer evident during the first pandemic year.

Many of the infection preventive measures implemented during the early phase of the pandemic were removed in 2021 and 2022. The incidence rates of infections returned to pre-pandemic levels in the period from March 2021 to March 2023, with an overall infection rate of 337 per 100 person-years (95% CI 315–363; *P* < 0.001 vs. first pandemic year). Increases in infection rates as compared to the first pandemic year were also specifically observed for respiratory infections (242 per 100 person-years, 95% CI 218–264), gastrointestinal infections (34 per 100 person-years; 95% CI 28–43), and Coxsackie virus infections (9 per 100 person-years; 95% CI 6–13). In particular, the respiratory infection rate from March 2021 to March 2023 was increased by 12% as compared to the pre-pandemic period (*P* = 0.02). The relationship between age and infection rates observed prior to the pandemic also returned for all infections, respiratory infections, gastrointestinal infections and Coxsackie virus infections.

### Incidence of islet autoantibodies in relation to the pandemic

**Fig. 2:**
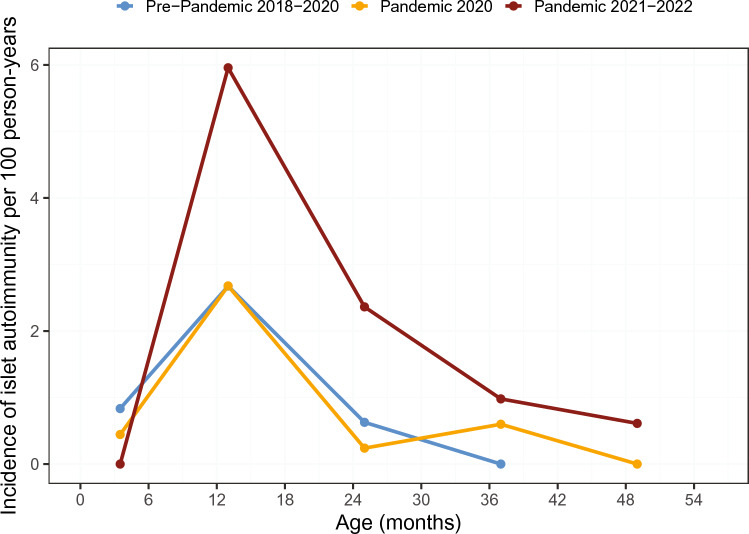
Incidence of Islet Autoantibodies in Children at Increased Risk for Type 1 Diabetes Before and During the COVID-19 Pandemic. Incidence of islet autoimmunity (cases per 100 person years) for the period 2018–02-07 to 2020–02-29 (pre-pandemic, blue line), 2020–03-01 to 2021–02-28 (pandemic 2020, orange line), and 2021–03-01 to 2023–02-28 (pandemic 2021–2022, brown line)

In the pre-pandemic period, the incidence of islet autoantibody seroconversion exhibited a previously reported peak around 12 months of age, with a maximum incidence rate of 2.7 (95% CI; 1.5–4.5) per 100 person-years occurring between 7 to 19 months of age (Fig. 2). During the first year of the pandemic, there was no change in the islet autoantibody incidence rate observed at this age (2.7 per 100 person-years; 95% CI 1.7–4.2) and across all ages. Islet autoantibody incidence rates in the second pandemic year were, however, increased with a peak incidence at 7 to 19 months of 6.0 per 100 person-years (95% CI 3.5–9.5; *P* = 0.01 vs. first pandemic year, *P* = 0.02 vs. pre-pandemic year). The incidence rate of islet autoantibodies before age 36 months was 1.6 (95% CI 1.0–2.4) per 100 person-years in the pre-pandemic period, 1.2 per 100 person-years (95% CI 0.8–1.9) in the first pandemic year (*P* = 0.46), and 3.4 per 100 person-years (95% CI 2.3–4.8) in the subsequent pandemic years (*P* = 0.005 vs. pre-pandemic year; *P* < 0.001 vs. first pandemic year).

## Discussion

Monitoring infection episodes in children from age 4 months to 40 months between 2018 and 2023 demonstrated a marked decline in infection rate across all ages in the first pandemic year with a rebound to pre-pandemic levels thereafter. The fall and return to pre-pandemic levels was observed for both respiratory and gastrointestinal infections and included infections attributed to Coxsackie viruses, which are associated with the development of islet autoimmunity and type 1 diabetes in childhood [7, 19–22]. Despite the dramatic decline in infections in the first pandemic year, the islet autoantibody incidence did not decline and increased in subsequent years when infection incidence rates returned to pre-pandemic levels.

The decline in reported infection rate in children during the initial year of the pandemic aligns with the implementation of measures aimed at curbing infection transmission in 2020 and is evidenced by the reported reduction in specific infections like influenza and RSV during the winter of 2020/2021. Additionally, in line with the less stringent measures adopted by Sweden as compared to other countries in the study, there was a more modest decline in reported infection rate in children enrolled in Sweden than in most other countries. A return to pre-pandemic infection rates was observed in the second and subsequent years of the pandemic. This aligns with the surge in infections observed during the 2021/2022 and 2022/2023 autumn and winter periods [23, 24] and the perturbed epidemiology of certain infections in children after the introduction of COVID-19 [25].

A key finding was the relationship between infection rates and the incidence rate of islet autoantibodies in the children. Despite a substantial reduction in infection rates in the first year of the pandemic, there was no corresponding decrease in islet autoantibodies within the cohort of children. This was unexpected, especially considering the decline in Coxsackie virus-associated infections. We have reported a notable temporal association between COVID-19 infection and the development of islet autoantibodies in these children and have postulated that COVID-19 had substituted for the associations between virus and islet autoimmunity from the pre-pandemic period [9]. In the subsequent years of the pandemic, with infection rates returning to pre-pandemic levels in 2021 to 2023, there was a simultaneous more than doubling of the islet autoantibody incidence rate. One explanation is that SARS-CoV-2, as a novel virus, has increased the susceptibility of developing islet autoantibodies in early childhood. Its introduction may have led to an overall increase in viral exposures that increase this susceptibility. Other explanations include an infection deficiency that led to less protection against infections that are associated with islet autoimmunity in the later pandemic years. Whether the increased islet autoantibody incidence will result in more cases of childhood type 1 diabetes cases depends on whether the observed increase was caused by an acceleration of islet autoantibody seroconversion during childhood or an actual increase in cases.

We also investigated trends in infection age to evaluate whether they align with the incidence rate of islet autoantibodies. The TEDDY study, which analyzed infection rates between 2006 and 2017, has previously reported a peak infection rate at around 1 year of age, followed by a subsequent decline, a pattern consistent with the peak age of islet autoantibody incidence [5]. However, our observations from 2018 to 2023 did not reveal a parallel age peak between infections and islet autoantibody incidence rates. While the peak islet autoantibody incidence rate in this study occurred at around 12 months of age, as previously reported [26], the peak infection rate was observed after 12 months, plateauing from around 18 months of age. These findings contradict the existence of a direct relationship between infection rate and the rate of islet autoimmunity. Instead, they suggest that infections are more likely to increase susceptibility to develop islet autoimmunity when they occur very early in life, a pattern demonstrated in the case of COVID-19 [9]. This interpretation aligns with the notion that the observed peak incidence of islet autoantibodies may be attributed to intrinsic features of the pancreatic islet and/or immune system at this age, rather than solely an abundance of diabetogenic exposures [2, 18].

The strength of our evaluation lies in the fact that infections were assessed within the framework of a clinical trial, and their monitoring was regularly conducted by local trial monitors, suggesting high data quality. However, there are limitations to consider. The pre-pandemic study period is relatively limited, raising uncertainty about the representativeness of infection rates from this period compared to data collected over a longer timeframe. Infections were solely recorded based on medical history and were not corroborated by biomarkers. It is well-established that actual infection episodes, as measured by antibodies, for example, can be significantly higher than those reported by families or detected through virus identification [27]. Therefore, the reported infection rates are likely to be underestimated. Country differences in reported infection rates were observed, and while some of these variations may be attributed to different practices in the first pandemic year, it is plausible that the disparities in overall rates and rates of specific infection groups also reflect differences in reporting likelihood and nomenclature used by families in various countries. Similarly, it is possible that the lower infection incidence rate observed in children with a first-degree family history of type 1 diabetes may potentially be attributed to heightened preventive measures against infections by parents or reporting bias. Our study was confined to the first 3 years of life. It has been shown that associations between virus infections and islet autoimmunity observed in early childhood may not persist at older ages [28]. Therefore, our findings may not be representative of later childhood and adolescence. Our study was performed in European countries, and it is unknown whether the findings can be generalized to other countries or to different ethnic and racial groups.

In conclusion, the analysis reveals a marked perturbation of early childhood infection epidemiology during the pandemic, concurrent to the introduction of COVID-19 to the community. This perturbation was followed by a significant increase in the incidence of islet autoimmunity in young children with an elevated genetic risk for type 1 diabetes. Further studies are warranted to continue the search for viruses that precede the onset of autoimmunity, and that determine the effect of vaccinations on the incidence of islet autoimmunity.

## Authors relationships and activities

The authors A-GZ and EB are inventors of a patent entitled ‘Method for determining the risk to develop type 1 diabetes’. MDS has been an investigator on behalf of the University of Oxford for clinical research funded or otherwise supported by vaccine manufacturers including Pfizer, AstraZeneca, GlaxoSmithKline, Novavax and MCM vaccines. He received no personal payment for this work. Since September 2022 he has been employed by Moderna Biomanufacturing Distributor UK, and holds equity in Moderna Inc. All other authors declare that there are no relationships or activities that might bias, or be perceived to bias, their work.

## GPPAD study group

See supplemental acknowledgements for details.

## Supplementary Information

Below is the link to the electronic supplementary material.

Supplementary file1 (DOCX 56 KB)

## Data Availability

Data will be available on submission of a signed transfer agreement; please email cc@gppad.org and the corresponding author.
