# Supplementary material for: Infection episodes and islet autoantibodies in children at increased risk for type 1 diabetes before and during the COVID-19 pandemic
Source: Infection. 2024 Jun 14;52(6):2465–73. doi: 10.1007/s15010-024-02312-y (PMC11621198; doi:10.1007/s15010-024-02312-y)
Supplement: Supplementary file 1 — Supplementary file1 (DOCX 56 KB) [file 15010_2024_2312_MOESM1_ESM.docx]

**SUPPLEMENT**

**Infection episodes and islet autoantibodies in children at increased risk for type 1 diabetes before and during the COVID-19 pandemic**

Ivo Zeller^1^, Andreas Weiss^1^, Stefanie Arnolds^1^, Katharina Schütte-Borkovec^1^, Sari Arabi^2,3^, Thekla von dem Berge^4^, Kristina Casteels^5,6^ Angela Hommel^2^, Olga Kordonouri^4^, Helena Elding Larsson^7,8^, Markus Lundgren^7,9^, Anne Rochtus^5,6^, Matthew D Snape^10^, Agnieszka Szypowka^11^, Manu Vatish^12^, Christiane Winkler^1^, Ezio Bonifacio^2,13^, Anette-Gabriele Ziegler^1,14,15^ for the GPPAD Study Group*

^1^Institute of Diabetes Research, Helmholtz Munich, German Center for Environmental Health, Munich, Germany

^2^Technische Universität Dresden, Center for Regenerative Therapies Dresden, Dresden, Germany

^3^Department of Pediatrics, University Hospital Carl Gustav Carus, Technische Universität Dresden, Dresden, Germany

^4^Kinder- und Jugendkrankenhaus AUF DER BULT, Hannover, Germany

^5^Department of Pediatrics, University Hospitals Leuven, Leuven, Belgium

^6^Department of Development and Regeneration, KU Leuven, Leuven, Belgium

^7^Unit for Pediatric Endocrinology, Department of Clinical Sciences Malmö, Lund University, Lund, Sweden

^8^Department of Paediatrics, Skåne University Hospital, Malmö/Lund, Sweden

^9^Department of Pediatrics, Kristianstad Hospital, Kristianstad, Sweden

^10^Oxford Vaccine Group, University of Oxford Department of Paediatrics, and NIHR Oxford Biomedical Research Centre, UK.

^11^Department of Paediatrics, Medical University of Warsaw, Warsaw, Poland

^12^Nuffield Department of Women’s & Reproductive Health, Oxford

^13^Paul Langerhans Institute Dresden of the Helmholtz Munich at University Hospital Carl Gustav Carus and Faculty of Medicine, TU Dresden, Germany

^14^Forschergruppe Diabetes e.V. at Helmholtz Munich, German Research Center for Environmental Health, Munich, Germany

^15^Forschergruppe Diabetes, School of Medicine, Klinikum rechts der Isar, Technical University Munich, Munich, Germany

***GPPAD Study Group**

GPPAD Coordinating Centre: Melanie Gündert, Stefanie Arnolds, Karina Blasius, Michaela Dilong,

Nadine Friedl, Gertrud Göppel, Florian Haupt, Martin Heigermoser, Maja Hergl, Bianca Höfelschweiger, Krisztian Kisfügedi, Nadine Klein, Stefanie Löbner, Rebecca Niewöhner, Marlon

Scholz, Lena Schwenker, Katharina Schütte-Borkovec, Andreas Weiß, José Maria Zapardiel Gonzalo,

Lorena Wendel

Belgium Clinical Centre: Kristina Casteels, Jasmin Paulus, Brontë Vrancken, Charlien Jannsen, Anne

Rochtus, An Jacobs, Hilde Morobé, Natalie Van den Driessche, Renka Van Heyste, Janne Houben,

Veerle Vanhuyse.

Germany, Dresden Clinical Centre: Ezio Bonifacio, Reinhard Berner, Sari Arabi, Lisa Barbknecht, Uta Ceglarec, Sevina Dietz, Franziska Ehrlich, Gita Gemulla, Zahra Gholizadeh, Raphael Hoffmann, Angela Hommel, Franziska Lange, Anja Loff, Peter Mirtschink, Robert Morgenstern, Anne Schille, Maike Sigg, Marc Weigelt, Andre Weise, Pauline Wimberger, Nicole Zubizarreta.

Germany, Hanover Clinical Centre: Olga Kordonouri, Thomas Danne, Laura Galuschka, Carolin

Kruse, Sarah Landsberg, Karin Lange, Erika Marquardt, Felix Reschke, Frank Roloff, Thekla von dem Berge, Jantje Weiskorn, Mareike Niemeyer, Bianca Schmidt

Germany, Munich Clinical Centre: Anette-G. Ziegler, Peter Achenbach, Evangelia Asvou, Melanie Bunk, Nicole Bücheler, Anna Hofelich, Elisabeth Huber, Stefanie Jacobson, Janna Jaud, Melina Kaiser, Alexandra Käßl, Benjamin Marcus, Annette Munzinger, Franziska Reinmüller, Katharina Sarcletti, Sarah Schmidt, Franka Teichgräber, Veronika Vollmuth, Merve Vurucu, Christiane Winkler

Poland Clinical Centre: Agnieszka Szypowska, Mariusz Ołtarzewski, Sylwia Dybkowska, Lidia

Groele, Dorota Owczarek, Katarzyna Popko, Adrianna Cieloch, Elżbieta Górska, Agnieszka Mroczek,

Beata Zduńczyk, Anna Zych, Wiktoria Czerwińska, Natalia Dziedzic

Sweden Clinical Centre: Helena Elding Larsson, Markus Lundgren, Daniel Agardh, Sofie Alström Mortin, Rasmus Bennet, Charlotte Brundin, Susanne Dahlberg, Lina Fransson, Malin Goldman-Tsubarah, Maria Hyllengren, Ida Jönsson, Marielle Lindström, Jessica Melin, Hannah Nenonen, Anita Ramelius, Kobra Rhamati, Falastin Salami, Hanna Samuelsson, Carina Törn, Ulrika Ulvenhag

United Kingdom, Oxford Clinical Centre: Rachel Besser, John A Todd, Matthew Snape, Sophia

Hawkins, Yama Farooq, Ian Smith, Helen Ratcliffe, Fenella Roseman, Hannah Robinson, Nazia Taj,

Sophie Vernon, Conor Whelan, Tabitha Wishlade, Manu Vatish

 Data Safety and Monitoring Board: Polly Bingley (University of Bristol, Bristol, UK), Ulrich Heininger (Division of Paediatric Infectious Diseases and Vaccinology, University Children's Hospital, Basel, Switzerland), Markus Pfirrmann (Institut für Medizinische Informationsverarbeitung, Biometrie und Epidemiologie (IBE), Ludwig-Maximilians-Universität, Munich, Germany), Wolfgang Rascher (Departmentof Pediatrics and Adolescent Medicine, Erlangen, Germany), Paul Turner (Nuffield Department of Medicine, Medical Science Division, Oxford, UK)

Beta-cell Autoantibody and Confirmatory Laboratory: Bristol Medical School, The University of Bristol, Bristol, UK

Genotyping Laboratory: Grace London, LGC Ltd., Hertfordshire, UK

Electronic Questionnaires/ Database/ Pharmacovigilance: PHARMALOG, GmbH, Ismaning, Germany

**Supplemental Table 1:** List of SNPs used to determine study eligibility and risk score calculation

| SNP | Gene, Allele, or Haplotype | Score weight for genotype or per allele |
| --- | --- | --- |
| **HLA class II** |  |  |
| rs17426593  rs2187668 rs7454108 | HLA DR4-DQ8/DR4-DQ8 | 3.15 |
|  | HLA DR3/DR4-DQ8 | 3.98 |
|  |  |  |
| rs3129889 | HLA *DRB1**1501 | Exclusion criteria for first degree relatives |
| rs1794265 | HLA *DQB1**0503 | Exclusion criteria for first degree relatives |
| **HLA class I** |  |  |
| rs1264813 | HLA A 24 | 0.43 |
| rs2395029 | HLA B 5701 | 0.92 |
| **Non-HLA SNPs** |  |  |
| rs2476601 | *PTPN22* | 0.76 |
| rs2816316 | *RGS1* | 0.16 |
| rs3024505 | *IL10* | 0.22 |
| rs1990760 | *IFIH1* | 0.16 |
| rs3087243 | *CTLA4* | 0.16 |
| rs10517086 | *C4orf52* | 0.19 |
| rs2069763 | *IL2* | 0.11 |
| rs6897932 | *IL7R* | 0.19 |
| rs3757247 | *BACH2* | 0.19 |
| rs9388489 | *C6orf173* | 0.14 |
| rs6920220 | *TNFAIP3* | 0.15 |
| rs1738074 | *TAGAP* | 0.05 |
| rs7804356 | *SCAP2* | 0.15 |
| rs4948088 | *COBL* | 0.17 |
| rs7020673 | *GLIS3* | 0.23 |
| rs12722495 | *IL2RA* | 0.47 |
| rs947474 | *PRKCQ* | 0.15 |
| rs10509540 | *RNLS/C10orf59* | 0.25 |
| rs689 | *INS* | 0.65 |
| rs4763879 | *CD69* | 0.06 |
| rs2292239 | *ERBB3* | 0.36 |
| rs3184504 | *SH2B3* | 0.24 |
| rs1465788 | *ZFP36L1* | 0.13 |
| rs17574546 | *RASGRP1* | 0.13 |
| rs3825932 | *CTSH* | 0.15 |
| rs12708716 | *CLEC16A* | 0.15 |
| rs4788084 | *IL27* | 0.20 |
| rs7202877 | *CTRB2* | 0.19 |
| rs2290400 | *ORMDL3* | 0.25 |
| rs7221109 | *CCR7* | 0.15 |
| rs45450798 | *PTPN2* | 0.09 |
| rs763361 | *CD226* | 0.12 |
| rs425105 | *PRKD2* | 0.21 |
| rs2281808 | *SIRPG* | 0.07 |
| rs3788013 | *UBASH3a* | 0.16 |
| rs5753037 | *RPS3AP51* | 0.15 |
| rs229541 | *IL2B* | 0.18 |
| rs5979785 | *TLR8* | 0.09 |
| rs2664170 | *GAB3* | 0.14 |

The risk score is calculated by multiplying the number of risk alleles (i. e. 0, 1 or 2 for each single SNP) with the weight assigned to each SNP and then summing up the weighted contributions of all SNPs plus an additive constant of 3.15 for infants who have the HLA DR4-DQ8/DR4-DQ8 genotype or 3.98 for infants who have the HLA DR3/DR4-DQ8 genotype. As an example, the risk score for a child with HLA DR4-DQ8/DR4-DQ8, homozygous for the risk allele of rs1264813 (weight 0.43), heterozygous for the risk allele of rs2395029 (weight 0.92), homozygous for the non-risk allele of rs2476601 (weight 0.76) and for all other SNPs in the genetic risk score is calculated as follows:

Risk score = 3.15 + (2 * 0.43) + (1 * 0.92) + (0 * 0.76) + 0 = 4.93

**Supplemental Table 2 MedDRA Low Level Terms and assigned categories**

| LLT-Code | Low Level Term | Category |
| --- | --- | --- |
| 10000687 | Acute bronchitis | Respiratory infection episodes |
| 10000823 | Acute laryngitis | Respiratory infection episodes |
| 10000827 | Acute laryngotracheitis | Respiratory infection episodes |
| 10066740 | Acute respiratory tract infection | Respiratory infection episodes |
| 10066743 | Acute rhinitis | Respiratory infection episodes |
| 10001076 | Acute sinusitis | Respiratory infection episodes |
| 10001257 | Adenoviral conjunctivitis | Respiratory infection episodes |
| 10001260 | Adenoviral upper respiratory infection | Respiratory infection episodes |
| 10060931 | Adenovirus infection | Respiratory infection episodes |
| 10001002 | Acute pharyngitis | Respiratory infection episodes |
| 10050547 | Angina tonsillaris | Respiratory infection episodes |
| 10004573 | Bilateral otitis media | Respiratory infection episodes |
| 10006448 | Bronchiolitis | Respiratory infection episodes |
| 10006451 | Bronchitis | Respiratory infection episodes |
| 10061736 | Bronchitis bacterial | Respiratory infection episodes |
| 10053160 | Bronchitis viral | Respiratory infection episodes |
| 10006469 | Bronchopneumonia | Respiratory infection episodes |
| 10084268 | COVID-19 | Respiratory infection episodes |
| 10008477 | Chest infection | Respiratory infection episodes |
| 10009851 | Cold | Respiratory infection episodes |
| 10009867 | Cold symptoms | Respiratory infection episodes |
| 10010106 | Common cold | Respiratory infection episodes |
| 10010741 | Conjunctivitis | Respiratory infection episodes |
| 10061784 | Conjunctivitis bacterial | Respiratory infection episodes |
| 10053983 | Corona virus infection | Respiratory infection episodes |
| 10051905 | Coronavirus infection | Respiratory infection episodes |
| 10011216 | Coryzal symptoms | Respiratory infection episodes |
| 10011224 | Cough | Respiratory infection episodes |
| 10011232 | Coughing | Respiratory infection episodes |
| 10011415 | Croup | Respiratory infection episodes |
| 10014011 | Ear infection | Respiratory infection episodes |
| 10072908 | Ear, nose and throat infection | Respiratory infection episodes |
| 10014909 | Enterovirus infection | Respiratory infection episodes |
| 10015929 | Eye infection | Respiratory infection episodes |
| 10016790 | Flu | Respiratory infection episodes |
| 10016796 | Flu-like illness | Respiratory infection episodes |
| 10048763 | Fluid in middle ear | Respiratory infection episodes |
| 10022000 | Influenza | Respiratory infection episodes |
| 10022002 | Influenza A virus infection | Respiratory infection episodes |
| 10023874 | Laryngitis | Respiratory infection episodes |
| 10063070 | Laryngitis bacterial | Respiratory infection episodes |
| 10023884 | Laryngotracheo bronchitis | Respiratory infection episodes |
| 10023898 | Laryngotracheobronchitis | Respiratory infection episodes |
| 10024968 | Lower respiratory tract infection | Respiratory infection episodes |
| 10062545 | Middle ear effusion | Respiratory infection episodes |
| 10065682 | Obstructive bronchitis | Respiratory infection episodes |
| 10033071 | Otitis | Respiratory infection episodes |
| 10033072 | Otitis externa | Respiratory infection episodes |
| 10033078 | Otitis media | Respiratory infection episodes |
| 10033079 | Otitis media acute | Respiratory infection episodes |
| 10033081 | Otitis media chronic | Respiratory infection episodes |
| 10033086 | Otitis media serous | Respiratory infection episodes |
| 10034835 | Pharyngitis | Respiratory infection episodes |
| 10057869 | Pharyngitis bacterial | Respiratory infection episodes |
| 10049140 | Pharyngotonsillitis | Respiratory infection episodes |
| 10035664 | Pneumonia | Respiratory infection episodes |
| 10060946 | Pneumonia bacterial | Respiratory infection episodes |
| 10050187 | Pseudocroup | Respiratory infection episodes |
| 10056752 | Purulent rhinorrhea | Respiratory infection episodes |
| 10039247 | RSV infection | Respiratory infection episodes |
| 10037993 | Reactive airways disease | Respiratory infection episodes |
| 10038700 | Respiratory infection | Respiratory infection episodes |
| 10062352 | Respiratory tract infection | Respiratory infection episodes |
| 10062106 | Respiratory tract infection viral | Respiratory infection episodes |
| 10049410 | Respiratory tract infection viral NOS | Respiratory infection episodes |
| 10039083 | Rhinitis | Respiratory infection episodes |
| 10067475 | Rhinopharyngitis | Respiratory infection episodes |
| 10052106 | Rhinosinusitis | Respiratory infection episodes |
| 10039296 | Runny nose | Respiratory infection episodes |
| 10084272 | SARS-CoV-2 infection | Respiratory infection episodes |
| 10039587 | Scarlet fever | Respiratory infection episodes |
| 10040753 | Sinusitis | Respiratory infection episodes |
| 10062660 | Sniffles | Respiratory infection episodes |
| 10041367 | Sore throat | Respiratory infection episodes |
| 10061372 | Streptococcal infection | Respiratory infection episodes |
| 10042182 | Streptococcal pharyngitis | Respiratory infection episodes |
| 10042186 | Streptococcal sore throat | Respiratory infection episodes |
| 10042189 | Streptococcal tonsillitis | Respiratory infection episodes |
| 10076988 | Subglottic laryngitis | Respiratory infection episodes |
| 10084451 | Suspected COVID-19 | Respiratory infection episodes |
| 10043520 | Throat infection | Respiratory infection episodes |
| 10044008 | Tonsillitis | Respiratory infection episodes |
| 10065235 | Tonsillitis bacterial | Respiratory infection episodes |
| 10046733 | URTI | Respiratory infection episodes |
| 10046299 | Upper resp tract infection | Respiratory infection episodes |
| 10046300 | Upper respiratory infection | Respiratory infection episodes |
| 10046306 | Upper respiratory tract infection | Respiratory infection episodes |
| 10046308 | Upper respiratory tract infection viral NOS | Respiratory infection episodes |
| 10047463 | Viral infection NOS | Respiratory infection episodes |
| 10047473 | Viral pharyngitis | Respiratory infection episodes |
| 10047474 | Viral pneumonia | Respiratory infection episodes |
| 10047482 | Viral upper respiratory tract infection | Respiratory infection episodes |
| 10011261 | Coxsackie viral infection | Respiratory infection episodes, Coxsackie infection episodes |
| 10016961 | Foot and mouth disease | Respiratory infection episodes, Coxsackie infection episodes |
| 10019124 | Hand, foot and mouth disease | Respiratory infection episodes, Coxsackie infection episodes |
| 10019936 | Herpangina | Respiratory infection episodes, Coxsackie infection episodes |
| 10066762 | Acute gastroenteritis | Gastrointestinal infection episodes |
| 10051226 | Campylobacter infection | Gastrointestinal infection episodes |
| 10014866 | Enteritis | Gastrointestinal infection episodes |
| 10058839 | Enteritis infectious | Gastrointestinal infection episodes |
| 10014893 | Enterocolitis | Gastrointestinal infection episodes |
| 10017888 | Gastroenteritis | Gastrointestinal infection episodes |
| 10059169 | Gastroenterocolitis | Gastrointestinal infection episodes |
| 10017964 | Gastrointestinal infection | Gastrointestinal infection episodes |
| 10039436 | Salmonella infection NOS | Gastrointestinal infection episodes |
| 10002509 | Angular cheilitis | Other types of infection |
| 10061663 | Atypical mycobacterial infection | Other types of infection |
| 10060945 | Bacterial infection | Other types of infection |
| 10004044 | Bacterial infection NOS | Other types of infection |
| 10061591 | Borrelia infection | Other types of infection |
| 10080463 | Boston exanthema | Other types of infection |
| 10006563 | Bullous impetigo | Other types of infection |
| 10007132 | Candida diaper rash | Other types of infection |
| 10008505 | Chickenpox | Other types of infection |
| 10009864 | Cold sore (herpetic) | Other types of infection |
| 10049319 | Cold sore mouth | Other types of infection |
| 10009865 | Cold sores | Other types of infection |
| 10011502 | Cryptosporidiosis infection | Other types of infection |
| 10011781 | Cystitis | Other types of infection |
| 10012502 | Dermatomycosis, unspecified | Other types of infection |
| 10014077 | EBV infection | Other types of infection |
| 10014881 | Enterobiasis | Other types of infection |
| 10015214 | Erythema infectiosum | Other types of infection |
| 10015586 | Exanthema subitum | Other types of infection |
| 10016284 | Febrile convulsion | Other types of infection |
| 10016290 | Febrile seizure | Other types of infection |
| 10049362 | Fever blister | Other types of infection |
| 10016560 | Fever convulsions | Other types of infection |
| 10016670 | Fifth disease | Other types of infection |
| 10017533 | Fungal infection | Other types of infection |
| 10017543 | Fungal skin infection | Other types of infection |
| 10018300 | Glandular fever | Other types of infection |
| 10019941 | Herpes infection | Other types of infection |
| 10019942 | Herpes labialis | Other types of infection |
| 10019948 | Herpes simplex | Other types of infection |
| 10019973 | Herpes virus infection | Other types of infection |
| 10021842 | Infection NOS | Other types of infection |
| 10021835 | Infection localised | Other types of infection |
| 10025169 | Lyme disease | Other types of infection |
| 10025226 | Lymphangitis | Other types of infection |
| 10027810 | Molluscum contagiosum | Other types of infection |
| 10079260 | Mononucleosis | Other types of infection |
| 10028015 | Mouth abscess | Other types of infection |
| 10061300 | Mycoplasma infection | Other types of infection |
| 10052057 | Neuroborreliosis | Other types of infection |
| 10030963 | Oral candidiasis | Other types of infection |
| 10061324 | Oral fungal infection | Other types of infection |
| 10031026 | Oral thrush | Other types of infection |
| 10062569 | Oxyuriasis | Other types of infection |
| 10034686 | Peritonsillar abscess | Other types of infection |
| 10034907 | Phlegmon | Other types of infection |
| 10035070 | Pinworm infection | Other types of infection |
| 10035071 | Pinworms | Other types of infection |
| 10037596 | Pyelonephritis | Other types of infection |
| 10039206 | Rochalimaea quintana infection | Other types of infection |
| 10039222 | Roseola | Other types of infection |
| 10039223 | Roseola infantum | Other types of infection |
| 10039511 | Scabies | Other types of infection |
| 10040062 | Septic arthritis NOS | Other types of infection |
| 10040555 | Shingles | Other types of infection |
| 10050048 | Sixth disease | Other types of infection |
| 10066409 | Staphylococcal skin infection | Other types of infection |
| 10068505 | Stomatomycosis | Other types of infection |
| 10043649 | Thrush | Other types of infection |
| 10043867 | Tinea corporis | Other types of infection |
| 10048762 | Tooth infection | Other types of infection |
| 10046848 | UTI | Other types of infection |
| 10046544 | Urinary infection | Other types of infection |
| 10046571 | Urinary tract infection | Other types of infection |
| 10054088 | Urinary tract Infektion bacterial | Other types of infection |
| 10046980 | Varicella | Other types of infection |
| 10046983 | Varicella zoster | Other types of infection |
| 10047441 | Viral exanthem | Other types of infection |
| 10047461 | Viral infection | Other types of infection |
| 10047476 | Viral rash | Other types of infection |
| 10063126 | Worms | Other types of infection |
| 10049080 | Yeast infection | Other types of infection |
| 10007135 | Candida nappy rash | Other types of infection |
| 10051998 | Febrile infection | Unknown febrile episodes |
| 10016558 | Fever | Unknown febrile episodes |
| 10016563 | Fever of unknown origin | Unknown febrile episodes |
| 10058698 | Intermittent fever | Unknown febrile episodes |
| 10070594 | PFAPA syndrome | Unknown febrile episodes |
| 10078900 | Subfebrile | Unknown febrile episodes |

**Supplemental Table 3 Unadjusted incidence rates per age group and period for total infection episodes and infection categories in children at increased risk for type 1 diabetes**

| Age Group | Incidence of infection episodes per 100 person years (95% CI) | | |
| --- | --- | --- | --- |
|  | Pre-Pandemic | Pandemic 2020 | Pandemic 2021-2022 |
| **Total infections** | | | |
| 4-9 months | 349/128;  272 (245-302) | 204/101;  202 (175-231) | 72/31;  232 (181-292) |
| 9-15 months | 540/170;  317 (291-345) | 398/216;  184 (166-203) | 370/126;  293 (264-324) |
| 15-21 months | 333/91;  364 (326-426) | 339/188;  180 (162-201) | 999/227;  441 (414-469) |
| 21-27 months | 133/34;  390 (326 - 462) | 207/132;  157 (137-180) | 1372/333;  412 (390-434) |
| 27-33 months | 22/5;  420 (263-636) | 110/83;  132 (108-159) | 1347/354  381 (360-401) |
| 33-40 months | 0/0; | 25/22;  113 (73-167) | 705/180;  391 (363-421) |
| **Respiratory infections** | | | |
| 4-9 months | 250/128;  195 (172-221) | 115/101;  114 (94-136) | 52/31;  167 (125 -219) |
| 9-15 months | 340/170;  200 (179-222) | 235/216;  109 (95-124) | 266/126;  211(186-238) |
| 15-21 months | 207/91;  227 (197-260) | 223/188;  119 (104-135) | 723/227;  319 (296-343) |
| 21-27 months | 89/34;  261 (210-321) | 139/132;  106 (89-125) | 966/333;  290 (272-309) |
| 27-33 months | 17/5;  324 (189-519) | 73/83;  88 (69-110) | 988/354;  279 (262-297) |
| 33-40 months | 0/0; | 19/22;  86 (52-135) | 535/180;  297 (272-323) |
| **Gastrointestinal infections** | | | |
| 4-9 months | 17/128;  13 (8-21) | 11/101;  11 (5-19) | 3/31;  10 (2-28) |
| 9-15 months | 49/170;  29 (21-38) | 22/216;  10 (6-15) | 42/126;  33 (24-45) |
| 15-21 months | 43/91;  47 (34-63) | 14/188;  7 (4-13) | 111/227;  49 (40-59) |
| 21-27 months | 25/34;  73 (47-108) | 13/132;  10 (5-17) | 171/333;  51 (44-60) |
| 27-33 months | 4/5;  76 (21-195) | 4/83;  5 (1-12) | 158/354;  45 (38-52) |
| 33-40 months | 0/0; | 0/22;  0 (0-17) | 80/180;  44 (35-55) |
| **Coxsackie virus infections** | | | |
| 4-9 months | 4/128;  3 (1-8) | 1/101;  1 (0-6) | 1/31;  3 (0-18) |
| 9-15 months | 15/170;  9 (5-15) | 0/216;  0 (0-2) | 13/126;  1 (5-18) |
| 15-21 months | 16/91;  18 (10-28) | 4/188;  2 (1-5) | 32/227;  14 (10-20) |
| 21-27 months | 7/34;  21 (8-42) | 1/132;  1 (0-4) | 51/333;  15 (11-20) |
| 27-33 months | 0/5;  0 (0-70) | 1/83;  1 (0-7) | 45/354;  13 (9-17) |
| 33-40 months | 0/0; | 0/22;  0 (0-17) | 32/180;  18 (12-25) |

**Supplemental Table 4 Change in total infection rate from Prepandemic to Pandemic 2020 in each country**

| **Country** | **Relative Rate** | **P value** | **Adj. P value** | **Mean Stringency Index**  **Jan 2020 – Jan 2021^[[1]](#footnote-2)^** |
| --- | --- | --- | --- | --- |
| Germany | 0.33 (0.17-0.61) | < 0.001 | < 0.001 | 81 |
| Belgium | 0.56 (0.39-0.81) | 0.002 | 0.01 | 74 |
| UK | 0.33 (0.17-0.62) | 0.003 | 0.02 | 80 |
| Poland | 0.80 (0.65-0.98) | 0.03 | 0.15 | 82 |
| Sweden | 0.72 (0.58-0.89) | 0.002 | 0.01 | 67 |

Nanda Mehak, Aashima, Sharma Rajesh. Review of COVID-19 epidemiology and public health response in Europe in 2020. Clin Epidemiol Glob Health 2021;12:100882.

Doi: https://doi.org/10.1016/j.cegh.2021.100882.

1. [↑](#footnote-ref-2)
